# Brackish habitat dictates cultivable Actinobacterial diversity from marine sponges

**DOI:** 10.1371/journal.pone.0176968

**Published:** 2017-07-10

**Authors:** Gregory A. Ellis, Chris S. Thomas, Shaurya Chanana, Navid Adnani, Emily Szachowicz, Doug R. Braun, Mary Kay Harper, Thomas P. Wyche, Tim S. Bugni

**Affiliations:** 1 Pharmaceutical Sciences Division, School of Pharmacy, University of Wisconsin-Madison, Madison, Wisconsin, United States of America; 2 Department of Medicinal Chemistry, University of Utah, Salt Lake City, Utah, United States of America; Universidad Autonoma Metropolitana, MEXICO

## Abstract

Bacterial communities associated with marine invertebrates such as sponges and ascidians have demonstrated potential as sources of bio-medically relevant small molecules. Metagenomic analysis has shown that many of these invertebrates harbor populations of Actinobacteria, many of which are cultivable. While some populations within invertebrates are transmitted vertically, others are obtained from the environment. We hypothesized that cultivable diversity from sponges living in brackish mangrove habitats have associations with Actinobacterial populations that differ from those found in clear tropical waters. In this study, we analyzed the cultivable Actinobacterial populations from sponges found in these two distinct habitats with the aim of understanding the secondary metabolite potential. Importantly, we wanted to broadly evaluate the potential differences among these groups to guide future Actinobacterial collection strategies for the purposes of drug discovery.

## Introduction

Drug resistant infectious diseases represent a growing threat to human health and medical practices such as organ transplant and surgery. Bacterial natural products have been the best source of drug leads for infectious disease, but without new sources and innovative methods, typical screening programs yield mostly known antibiotics leading to an unsustainable discovery paradigm. One solution is to use bacteria from marine habitats, which have shown great promise as sources for new molecular scaffolds due to their bacterial diversity. Within marine habitats, culture independent techniques have shown that marine invertebrates maintain diverse microbial communities and are therefore an attractive location to search for sources of novel natural products. For example, marine sponges are filter feeding sessile animals that maintain stable associations with diverse bacteria.[1–3] Marine sponges have been important sources of medically relevant small molecules such as the anticancer agent halichondrin B, which served as a lead for the approved agent Halaven.[4–6] Interestingly, many of the small molecules found in whole animals are made by symbiotic bacteria highlighting the potential of sponge associated bacteria for the purposes of drug discovery.[7, 8] While it is known that marine sponges harbor cultivable Actinobacterial populations—a phylum known for its natural product producing proclivity—little is known about whether those populations differ in sponges from drastically different habitats.[9, 10] Knowledge regarding the bacterial diversity of these habitats could be crucial for informing collection strategies for natural product producing bacteria. Therefore, we sought to evaluate differences broadly between brackish habitats and tropical reef type habitats within the context of using bacteria for antibiotic discovery.

Two factors likely influence marine sponge-associated bacterial populations: habitat and host identity. Since sponges acquire symbiotic bacteria through vertical transmission as well as acquisition from the environment, habitat likely shapes aspects of the microbiome of sponges.[2, 11, 12] Yet, it has also been shown that sponge bacterial communities appear to reflect the host identity.[13] Given the change in environment, and that sponges inhabiting brackish habitats in Florida can differ from those found in the Florida Keys, the microbial communities should also be different. Cultivable Actinobacteria represent a minor component of the sponge microbiome and therefore will likely not correlate with host identity but rather environmental conditions, yet they are of specific interest to us given their history of natural product production. As such, the studies presented here were not aimed at discerning how habitat influences the full sponge microbiome per se, but rather to look from a broader perspective to identify groups of associated microorganisms, likely to have antibiotic producing potential, that were different between the two habitats. We hypothesized that Actinobacteria associated with sponges from brackish habitats might be more similar to terrestrial bacteria and that some phylogenetic groups may only be present in certain habitats. If so, the metabolites produced by bacteria associated with sponges from the two habitats might also be different indicating potential collection strategies for antibiotic discovery.

In order to evaluate the relatedness of microorganisms from each habitat, we used genomics as well as secondary metabolite analysis using untargeted LCMS-based metabolomics methods similar to those previously published for strain selection.[14, 15] We analyzed metabolite profiles using a variety of methods to understand the relationship among extract profiles from the two environments. As has been noted in a recent review, analysis of large datasets by PCA can be problematic.[16] Essentially, PCA fails to provide meaningful relationships when the intragroup variance is large relative to overall variance. Therefore, herein we explored the combined use of principal component analysis (PCA), hierarchal clustering (HCA), and partial least squares discriminate analysis (PLS-DA) to understand how habitat affects both phylogenetic and molecular diversity. Together, these studies would begin to elaborate patterns in cultivable microorganisms and their associated metabolites.

While these statistical methods have been used to a certain degree, studies thus far have been focused on small groups of bacteria. Extensive review of the literature reveals that nobody has yet applied these methods to the large numbers of samples detailed in our study. Enabling the metabolomics analyses of large numbers of strains requires a combination of methods to make sense of the data. Additionally, the application of metabolomics to understand differences between habitats has not been done.[17–20] In the end, we aimed to understand how sampling two different habitats affects chemical and phylogenetic diversity, and importantly if these two are related.

## Materials and methods

### Sponge collection and bacterial cultivation

To collect marine sponges in Florida, a fishing license was required. Both collectors, Tim S. Bugni and Navid Adnani, purchased fishing licenses. To ensure the collections did not violate local laws, we coordinated our collections with the Smithsonian Marine Station. Our study did not involve endangered or protected species. Vertebrates were not collected in our study.

Ten “brackish” sponge specimens were collected on August 7, 2013 near Stan Blum State Park boat launch (27°28’45.7”N, 80°18’42.8”W) in Florida, USA. Eleven “clear tropical” sponge specimens were collected on August 8, 2013 along the shore near the Keys Marine Lab, Florida, USA (24°49’35.8”N, 80°48’50.0”W). Sponge specimens included in this study, S1 Table, were taxonomically identified by Mary Kay Harper (University of Utah). Voucher specimens are housed at the University of Wisconsin-Madison. For cultivation, sponge samples (1 cm^3^) were ground in 500 μL sterile seawater and then diluted by addition of 500 μL sterile seawater. Subsequently, 400 μL of diluted sponge sample was added to 200 μL of sterile seawater and 100 μL was plated using a sterile L-shaped spreader. Dilutions were separately plated on six media supplemented with artificial seawater: ISP2, R2A, ISP3, Gauze 1, HV, Bonito (1g ground bonito flakes, 1g glucose, 1g peptone, 0.5g KH_2_PO_4_, 0.5g NH_4_Cl, and 7.5g agar, in 500 mL diH_2_O).[21–25] Each medium was supplemented with 50 μg/mL cycloheximide, 25 μg/mL nystatin, and 25 μg/mL nalidixic acid. HV medium was additionally supplemented with 25 μg/mL gentamicin. Plates were incubated at 28°C and colonies were isolated over the course of two months.

### DNA isolation and sequencing

Cultures (10 mL) were grown in 25 × 150 mm culture tubes in DSC media (50% artificial seawater (ASW), 5 g/L soluble starch, 10 g/L dextrose, 5 g/L peptone, and 5 g/L yeast extract in diH_2_O). Genomic DNA was extracted using the UltraClean Microbial DNA Isolation kit (Mo Bio Laboratories, Inc., Carlsbad, CA, USA). 16S rDNA genes were amplified with the primers 8-27F (5’ to 3’ GAGTTTGATCCTGGCTCAG) and 1492R (5’ to 3’ GGTTACCTTGTTACGACTT).

### Phylogenetic analysis

The phylogenetic tree (S4 Fig) was constructed using Clustal Omega.[26] The tree was viewed using iTOL.[27]

### Fermentation of strains for LCMS profiling

Seed cultures (10 mL) were grown in 25 × 150 mm culture tubes in modified ASW-A (5g soluble starch, no CaCO_3_, in 50% artificial sea water) media until turbid at 200 RPM at 28°C. Erlenmeyer flasks (125 mL) containing 25 mL of ASW-A (20g soluble starch, 10g glucose, 5g peptone, 5g yeast extract, 5g CaCO_3_, per liter of artificial seawater) were inoculated with 1.25 mL of seed culture and shaken at 200 rpm at 28°C for at least seven days (for *Streptomyces* strains) or fourteen days (for Micromonosporaceae). For Micromonosporaceae, 70 g/L Diaion^®^ HP-20 resin (Supelco) was added as an adsorbent. Iron sulfate and EDTA were added to Micromonosporaceae to a final concentration of 50 μM iron sulfate and 50 μM EDTA.

### Sample preparation of *Streptomyces* sp. for UHPLC/HRESI-TOF-MS

Two aliquots (1.5 mL each) were removed from cultures and stored at -80°C until use. Before processing, aliquots were thawed, transferred to a clean microcentrifuge tube (1.7 mL), and centrifuged at 10,000 rpm for 1 min. The supernatant (1 mL) was transferred into a clean vial and placed on a Gilson GX-271 liquid handling system. A total of 900 μL of supernatant was subjected to automated solid-phase extraction (SPE) (Biotage: EVOLUTE^®^ ABN, 25 mg sorbent mass, 1 mL reservoir volume), washed with Milli-Q H_2_O (1 mL) to help remove media components/ primary metabolites, and eluted with MeOH (1 mL) directly into an LCMS-certified vial.

### Sample preparation of Micromonosporaceae (*Micromonospora*, *Verrucosispora*, and *Solwaraspora* spp.) for UHPLC/HRESI-TOF-MS

The contents of each flask (25 mL) were transferred to 50 mL conical tubes. Each flask was rinsed with 15 mL Milli-Q H_2_O and added to the 50 mL conical tubes. Tubes were centrifuged for 5 min at 4,500 rcf at ambient temperature, and the supernatant was discarded. To remove media components, 15 mL Milli-Q H_2_O was added to each tube containing cells and resin followed by vortexing for 10 s. Conical tubes were then centrifuged for 5 min at 4,500 rcf at ambient temperature. The supernatant was decanted and discarded. The wash protocol was repeated twice to ensure removal of the majority of media components. For extraction, 15 mL MeOH was added to each tube. Each sample was vortexed for 10 s, let sit 5 min, vortex mixed for 10 s, and then let to sit for approximately 2 h to let insoluble material settle. A total of 5 mL of supernatant was transferred into a 20 mL scintillation vial and dried using a centrifugal concentrator. Dried samples were stored at -80°C until further processing. To process extracts, MeOH was added (150 μL) to each vial, sonicated for 5 min, then diH_2_O was added (1350 μL) and sonicated for 5 min (final solution of 1.5 mL was MeOH:diH_2_O 10:90). Sample was transferred to a clean vial and placed on a Gilson GX-271 liquid handling system. A total of 200 μL of extract was subjected to automated SPE (Thermo Scientific, Waltham, MA, USA: HyperSep^™^ C18, 50 mg bed weight, 1 mL column capacity), washed using Milli-Q H_2_O (1 mL) to remove media components/primary metabolites, and eluted with MeOH:Milli-Q H_2_O (95:5; 1 mL) directly into an LCMS-certified vial. C18 was selected to minimize carryover of lipids from the extract into LCMS samples (versus ABN for *Streptomyces* sp.).

### UHPLC/HRESI-TOF-MS analysis

LCMS data were acquired using a Bruker MaXis^™^ 4G ESI-QTOF mass spectrometer coupled with a Waters Acquity UPLC system operated by Bruker Hystar software. RP chromatography was performed using a C18 column (Phenomenex Kinetex 2.6 μm, 2.1 mm × 100 mm) operated with a flow rate of 0.3 mL/ min. A gradient of MeOH and H_2_O (containing 0.1% formic acid) started from MeOH/ H_2_O (10%:90%), followed by a linear gradient to reach MeOH/ H_2_O (97%:3%) in 12 min, and held for 3.5 min at MeOH/ H_2_O (97%:3%). Full scan mass spectra (*m/z* 150−1550) were detected using (+)-ESI mode. The mass spectrometer was operated using the following parameters from 0–15.5 min: capillary, 4.5 kV; nebulizer pressure, 1.2 bar; dry gas flow, 8.0 L/min; dry gas temperature, 205°C; rolling average, 2; scan rate, 2 Hz. For automatic internal calibration, tune mix (Agilent, ESI-L low concentration) was introduced through a divert valve at the end of each chromatographic run.

### Data processing and data modeling

Bruker Data Analysis 4.2 was used for analysis of LCMS chromatograms. Finding molecular features, bucketing LCMS data, and PCA was performed using Bruker ProfileAnalysis 2.1. Finding molecular features was applied to LCMS line spectra acquired in positive ion mode data under these parameters: S/N threshold: 5; correlation coefficient threshold: 0.7; minimum compound length: 10 spectra; smoothing width: 1; bucketing basis: M+H. The bucket generation was performed under the following parameters: the LCMS data sets were evaluated in a time range from 120 s to 840 s and in a mass range from *m/z* 200 to 1500. Advanced bucketing was employed using Δ RT = 20 s and Δ *m/z* = 0.02 Da as parameters. Sum of bucket values was applied for normalization in this study, and Pareto scaling was applied.

Unsupervised hierarchal clustering plots were constructed in Profile Analysis 2.1. The distance measure was “correlation” and the linkage method was “average”. Pareto scaling was also applied. For PLS-DA, the “bucket table” from ProfileAnalysis 2.1 was exported as a.TXT file. Due to column limitations in MS Excel and LibreOffice Calc, the data was pre-processed using a simple R script. The output CSV file was then uploaded to metaboanalyst.ca using their “Statistical Analysis” workflow.[28–32] The data were processed as a “Spectral bin” file with samples in columns (unpaired). The data were filtered using standard deviation and then normalized by sum, and Pareto scaling was applied. The plots generated were exported as PNG files from the website using their interface.

### Antibacterial assay

Extracts were tested for antibacterial activity against *Bacillus subtilis* and *Escherichia coli*. For *Streptomyces* strains, LCMS vials post-SPE and post-LCMS were dried under vacuum and stored at -80°C. Prior to screening, 50 μL DMSO was added to each sample and sonicated for 5 min. For Micromonosporaceae, the extract remaining after SPE, was dried under vacuum and stored at -80°C. Prior to screening, 200 μL DMSO was added to each sample and sonicated for 10 min. Gentamycin (20 μg/mL) dissolved in Milli-Q H_2_O was used as the positive control. DMSO was used as the negative control. For the assay, *B*. *subtilis* and *E*. *coli* starter cultures (10 mL) were grown in 25 × 100 mm culture tubes in Luria broth (LB) at 37°C at 200 rpm overnight. Cation-adjusted Mueller-Hinton Agar (CAMHA) (1.5g soluble starch, 17.5g casein, 3g beef extract, 15g agar, 12.5mg Mg^2+^, 25mg Ca^2+^, in 1 L diH_2_O) was prepared and maintained at 50–60°C in a water bath. CAMHA (200 mL) was then inoculated with 4 mL of either *B*. *subtilis* or *E*. *coli* starter culture, gently swirled, and pipetted into 4 sterile OmniTrays (40 mL) (Thermo Scientific Nunc, Rochester, NY, USA). Once the agar had cooled and solidified, 5 μL of each sample or control was applied in a 96-spot array, with the first column used as negative control (8 spots) and the last column used as positive control (8 spots). The plates were incubated at 37°C overnight. Plates were then analyzed and recorded using an Epson Scanner. Bioactive samples were identified visually through zones of inhibition on the plate. All samples were tested in duplicate.

## Results and discussion

In part, we wanted to evaluate the diversity and biomedical potential of sponge-associated Actinobacteria from brackish habitats. Ten sponges were collected by snorkeling in mangrove habitats between the Atlantic Ocean and Indian River in Florida that represented the “brackish” sponges. Eleven sponges were collected by snorkeling in the Florida Keys, and those represented the “clear tropical” sponges. Six distinct media were used to target phylogenetically diverse Actinomycetes. A total of fifteen different genera were isolated from the two habitats. Given that the environment within the mangrove area was high in silt and suspended particles, we anticipated that these samples would yield higher numbers of bacterial isolates. While some of these would likely be considered environmental isolates rather than true sponge associates, we made no attempt to distinguish. These studies were not aimed at determining anything about symbiosis, but aimed at understanding how habitat affects outcomes for a drug discovery program, namely in terms of biological and chemical diversity. Not surprising, we cultivated and purified more bacterial strains from the brackish habitat (176 isolates) compared to the clear tropical habitat (94 isolates). Two important points came from this analysis: 1) the clear tropical habitat yielded more strains that have been associated with specificity for the marine environment while the brackish habitat yielded more strains that would classically be considered terrestrial; and 2) the brackish samples yielded more bacterial diversity.

### Phylogenetic diversity

**Fig 1:**
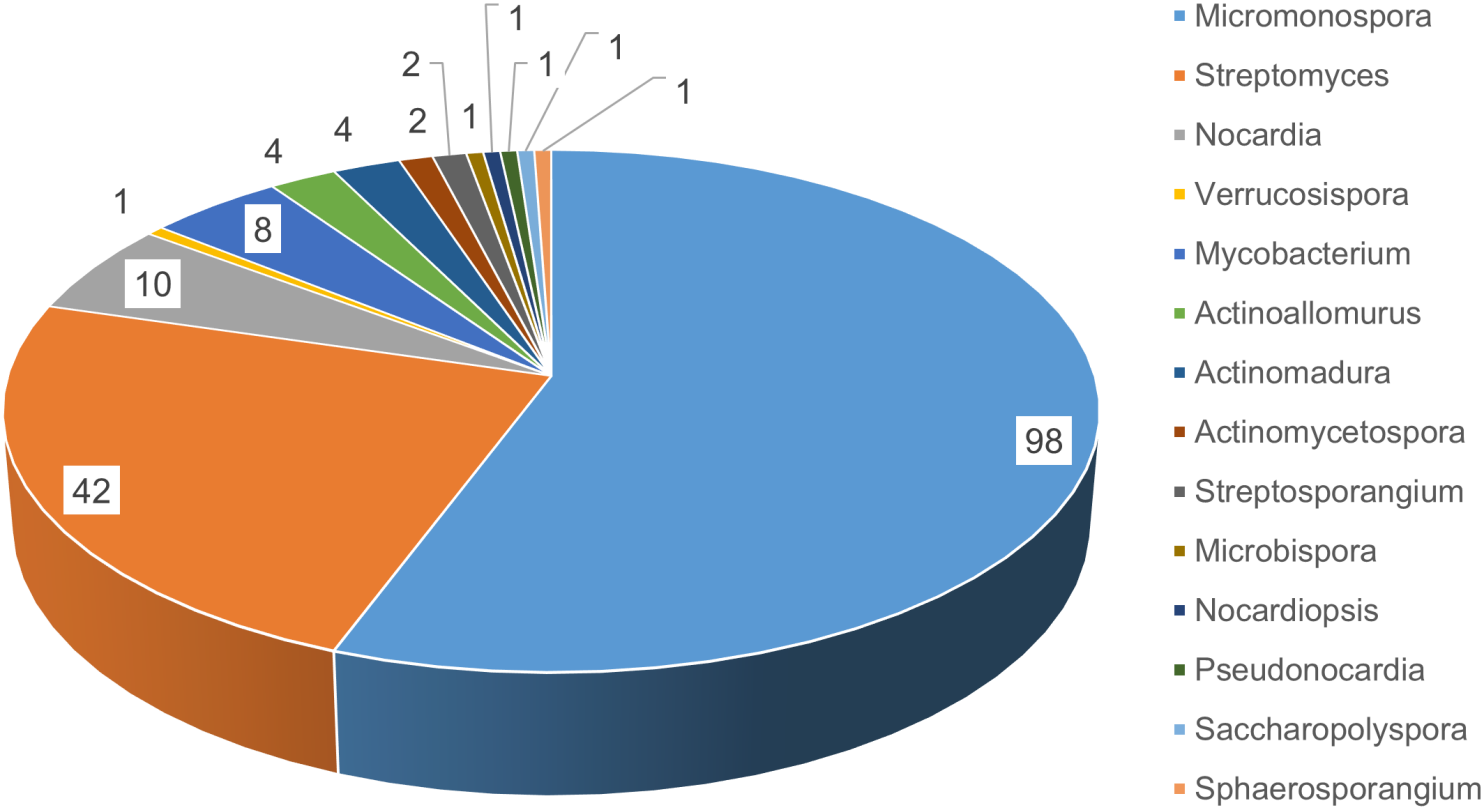
Actinobacteria genera isolated from brackish water sponges.

**Fig 2:**
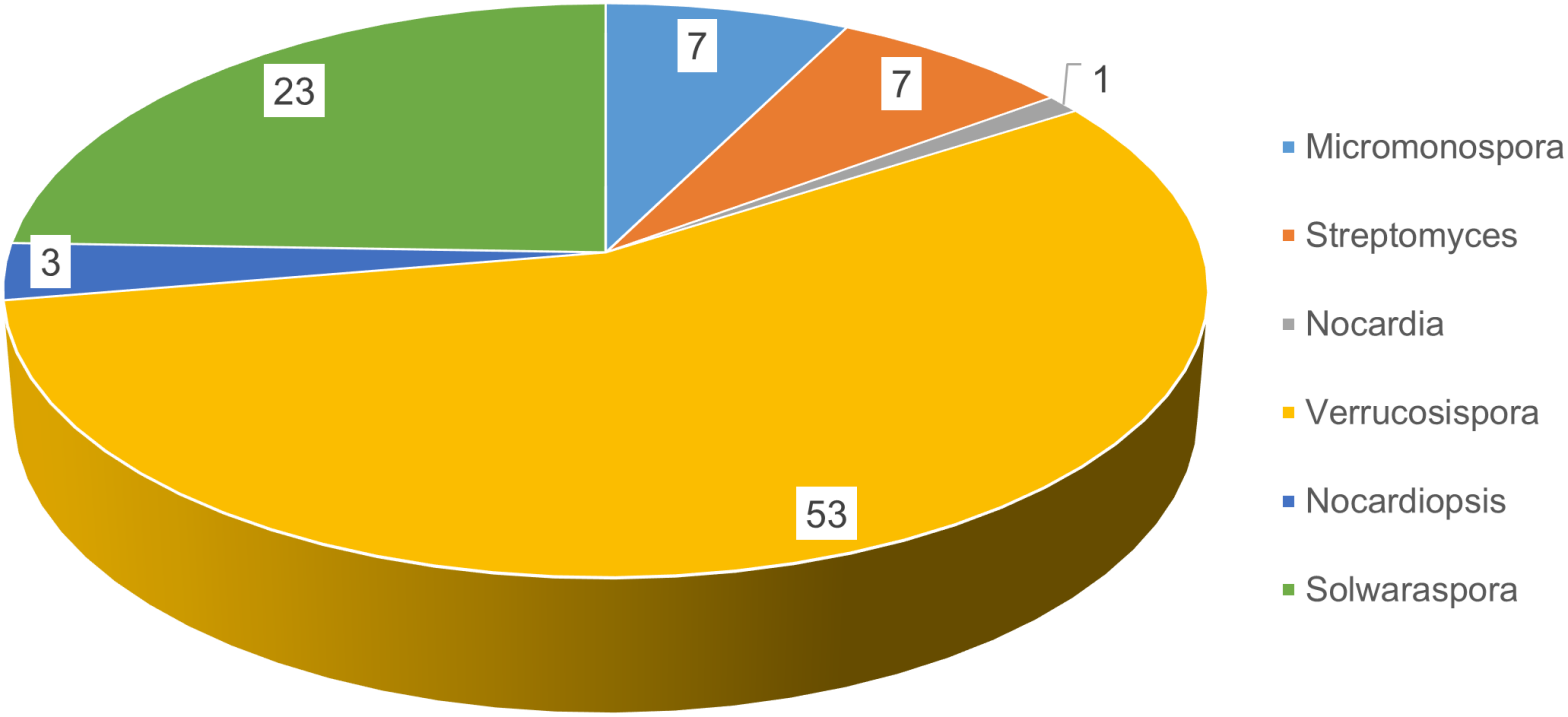
Actinobacteria genera isolated from clear tropical waters.

**Fig 3:**
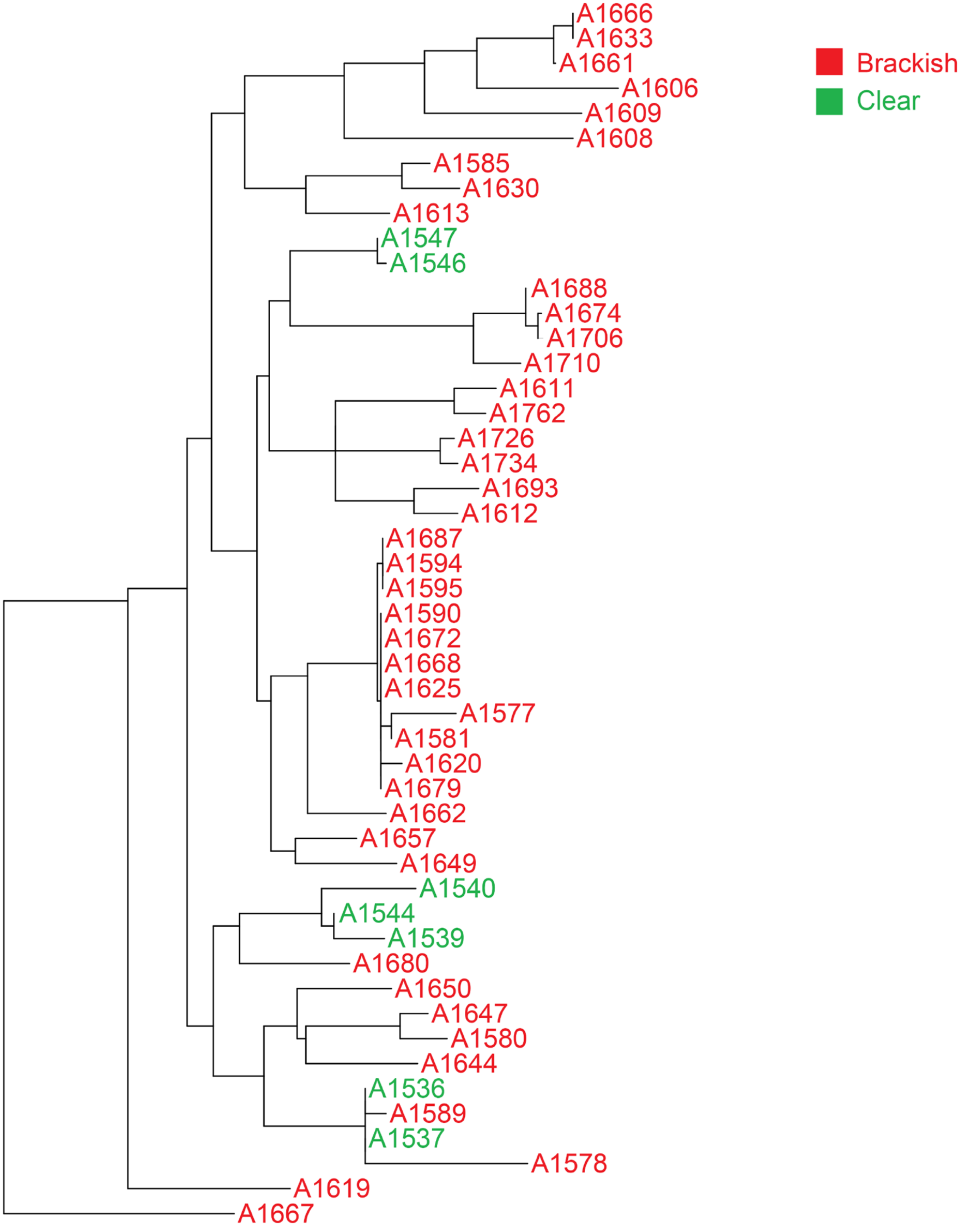
Phylogenetic tree of *Streptomyces* spp.

**Fig 4:**
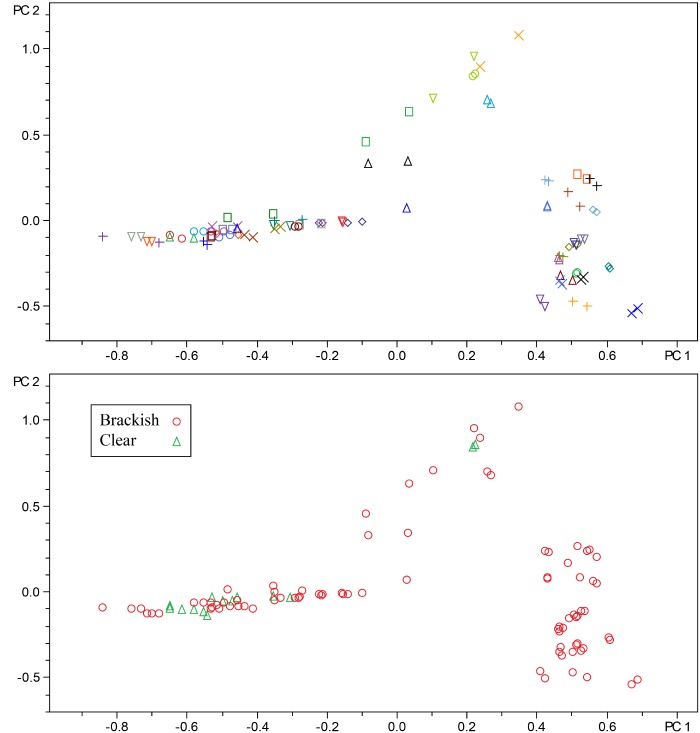
PCA of streptomyces spp. The top figure highlights strains in biological duplicate as denoted by the shapes and colors. The bottom figure distinguishes between brackish/clear isolates.

To evaluate phylogenetic diversity, we used 16S sequencing. What was particularly striking was the number of *Micromonospora* spp. cultivated from the brackish water sponges (total 98) compared to the clear tropical samples (total 7) (Figs 1 and 2). Additionally, *Streptomyces* spp. were also primarily cultivated from the brackish sponges. Phylogenetic comparison of the *Streptomyces* spp. that were cultivated from the clear tropical habitat showed that they were somewhat distinct (Fig 3), but metabolomics indicated that their laboratory molecular repertoire was not different (Fig 4 and S1 Fig). The predominant genera, however, for the clear tropical sponges were *Verrucosispora* (total 53) and *Solwaraspora* (total 23), unique and proposed genera that have been defined as having proclivity for the marine environment.[33–35] Overall, analyses by 16S sequencing showed specific differences in the cultivable diversity between the two habitats that were highly significant in terms of collection strategies.

### Metabolic diversity

While the brackish habitat was more prolific in terms of isolate numbers, we wanted to assess the metabolic diversity of those isolates in terms of their phylogenetic diversity. In particular, we wanted to assess the metabolite similarities and differences between bacteria from the two habitats with the goal of directing future collection strategies. We used LCMS-based metabolomics to assess metabolites, focusing on the four most abundant genera: *Streptomyces* spp., *Micromonospora* spp., *Solwaraspora* spp., and *Verrucosispora* spp.

For evaluating secondary metabolite diversity, extracts were made and analyzed using UHPLC-MS. To ensure reproducibility, we sampled *Streptomyces* spp. colonies in duplicate and evaluated the metabolite variance among strains using PCA (Fig 4). Two important points can be drawn from Fig 4 that directed analysis of the other strains. First, the reproducibility was excellent. Second, environmental replicates for most strains were present within the cultivated bacteria; in other words, most strains were independently isolated repeatedly, which correlates with the phylogenetic analysis. Therefore, we did not analyze replicates for other bacteria with the idea that these techniques are a quick screen and that anything particularly unique could be investigated further. For the purposes of drug discovery, we tend to focus on the presence of a putatively unique metabolite found in only one strain rather than concentration differences of identical metabolites over several strains. As such, we used Pareto scaling in all analyses to reduce the effect of ion intensity.

PCA analysis of the *Streptomyces* spp. showed that the isolates from the sponges collected from tropical reef habitats did not add additional chemical diversity. With respect to a drug discovery program, this would indicate that the brackish habitat would be the better choice for *Streptomyces* spp. and that with a combined collection between both habitats, it would not be necessary to isolate *Streptomyces* from sponges collected in the clear tropical habitat. Overall, the metabolomics show that the brackish environment yielded more chemically diverse or unique strains compared to the clear water sponges.

**Fig 5:**
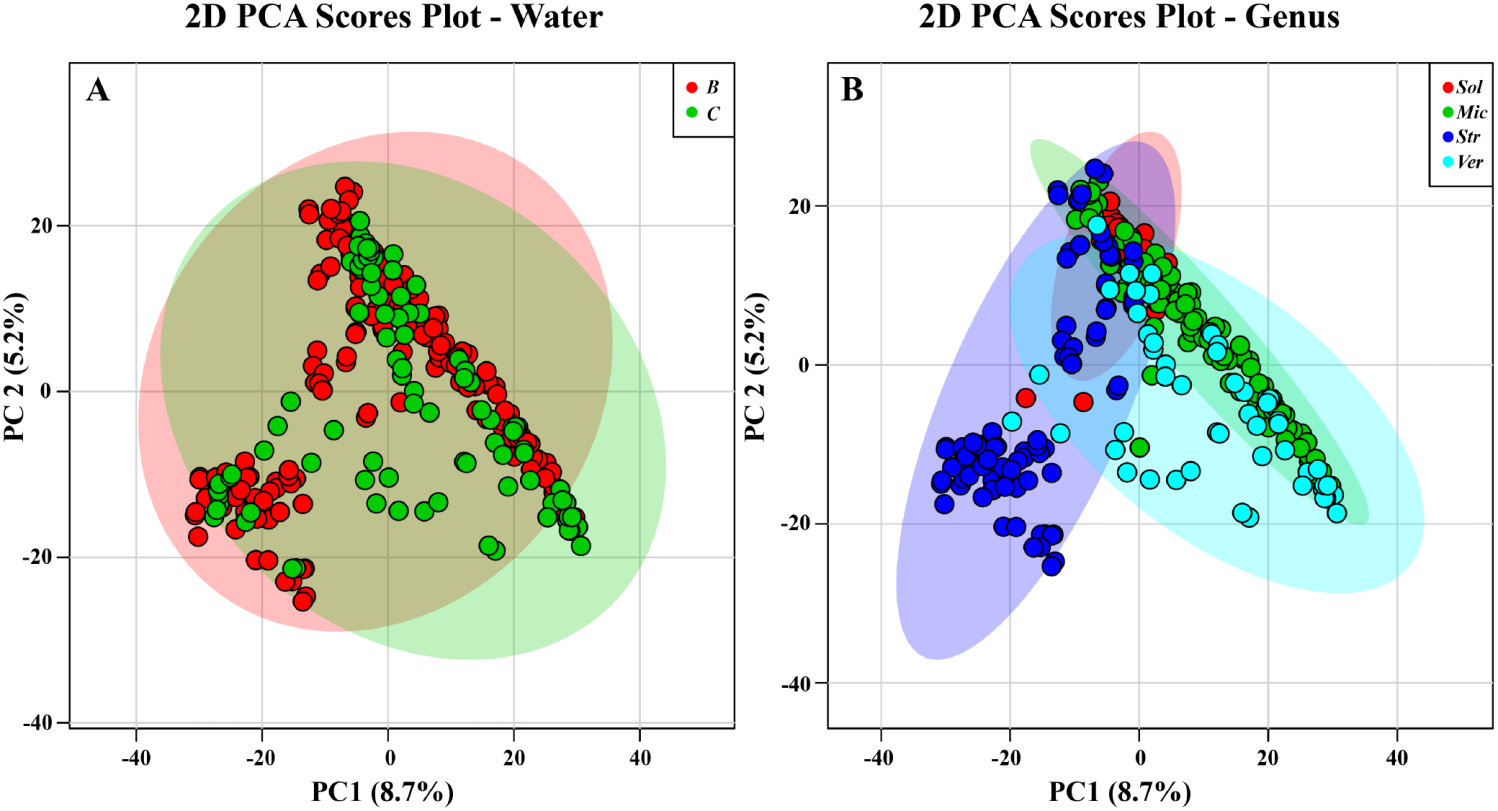
PCA of all bacterial strains. 95% confidence intervals are indicated by shaded area. (A) Samples are indicated based on clear [C] or brackish [B] water collection sites. (B) Samples are indicated based on bacterial genus: *Solwaraspora* [Sol], *Micromonospora* [Mic], *Streptomyces* [Str], or *Verrucosispora* [Ver]. Note: *Streptomyces* spp. were processed differently than the other three genera.

Having demonstrated that brackish waters show more diversity for *Streptomyces*, we next sought to extend our analyses to the other most abundant genera. Analysis of all profiles by PCA indicated some distinctions between the two environments, but overall, the dataset was likely too large for PCA to show appropriate relationships. For example, in Fig 5A, groups were identified solely based on clear versus brackish while in Fig 5B, groups were identified based on genus. In both figures, 95% confidence intervals were defined for the groups. While differences could be observed between the two groups, an immense amount of overlap made it difficult to draw any conclusions.

**Fig 6:**
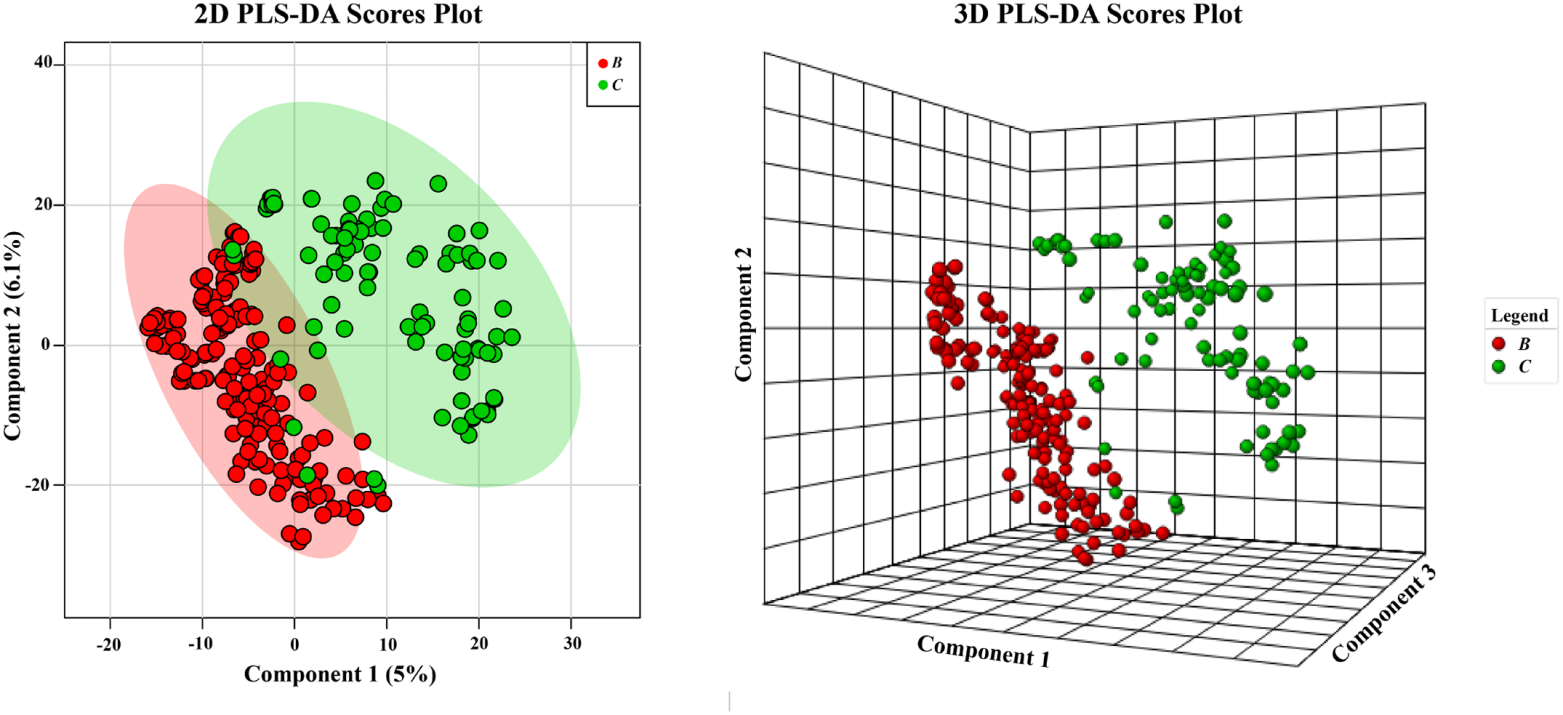
PLS-DA analysis of strains based on water. Samples are indicated based on clear [C] or brackish [B] water collection sites. 95% confidence intervals are indicated by shaded area. Note: *Streptomyces* spp. were processed differently than the other three genera.

Therefore, we analyzed the data sets using PLS-DA to help visually clarify differences among the groups. Mainly, we wanted to examine class structure between the two environments and determine what was driving the discrimination among groups. As shown in Fig 6, the separation of two groups can be observed using PLS-DA in contrast to the original PCA analysis in Fig 5. However, we made no attempt for cross validation, but instead used additional analyses of the groups by HCA and PCA to further investigate the profiles. Importantly, subsequent PCA shows that overlap in the PLS-DA tends to be driven by genera that were not common in one environment, but common in the other. For example, *Streptomyces* spp. from the clear tropical habitat were relatively few, but had metabolite profiles similar to the many *Streptomyces* spp. from the brackish environment (vide infra).

**Fig 7:**
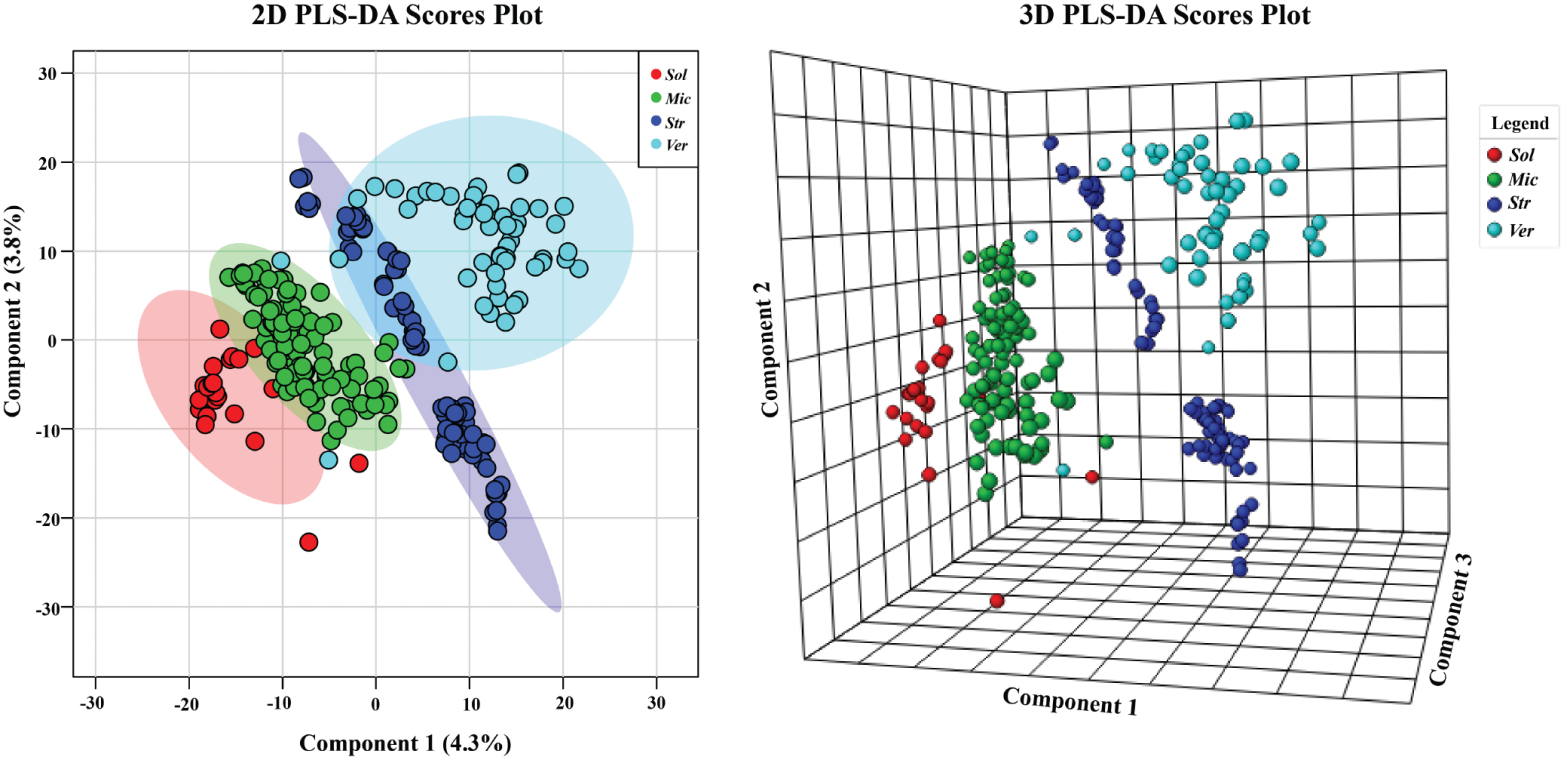
PLS-DA where groups were based on the bacterial genus. Samples are indicated based on bacterial genus: *Solwaraspora* [Sol], *Micromonospora* [Mic], *Streptomyces* [Str], or *Verrucosispora* [Ver]. 95% confidence intervals are indicated by shaded area. Note: *Streptomyces* spp. were processed differently than the other three genera.

The dependence of grouping was more apparent in PLS-DA when classification by genus was used (Fig 7). What was particularly interesting was that *Solwaraspora* spp. grouped closely with *Micromonospora* spp. While *Solwaraspora* has not been defined as a unique genus, they have distinguishing features in the lab, and we have isolated novel small molecules from them.[35, 36] Mainly, they grow much slower and have distinguishable visual characteristics on plates. Due to this similar grouping and our interest in *Solwaraspora*, we next used PLS-DA to ask questions about if *Solwaraspora* spp. belonged to the *Micromonospora* spp. group. PLS-DA shows that while *Solwaraspora* and *Micromonospora* overlap, they are also somewhat distinct, confirming what we have noticed in the lab. While *Solwaraspora* is not a formally recognized genus, we chose to distinguish members of that group from *Micromonspora* spp. due to distinguishable culture phenotypes including slow growth. In terms of metabolomics, *Solwaraspora* tends to group together suggesting that it is metabolically different, which could translate to novel small molecules. Since it can be distinguished and appears to produce different suites of molecules compared to the *Micronospora* spp. in this study, we felt that provided sufficient justification to note the distinction.

### Understanding metabolite diversity within each bacterial genus

**Fig 8:**
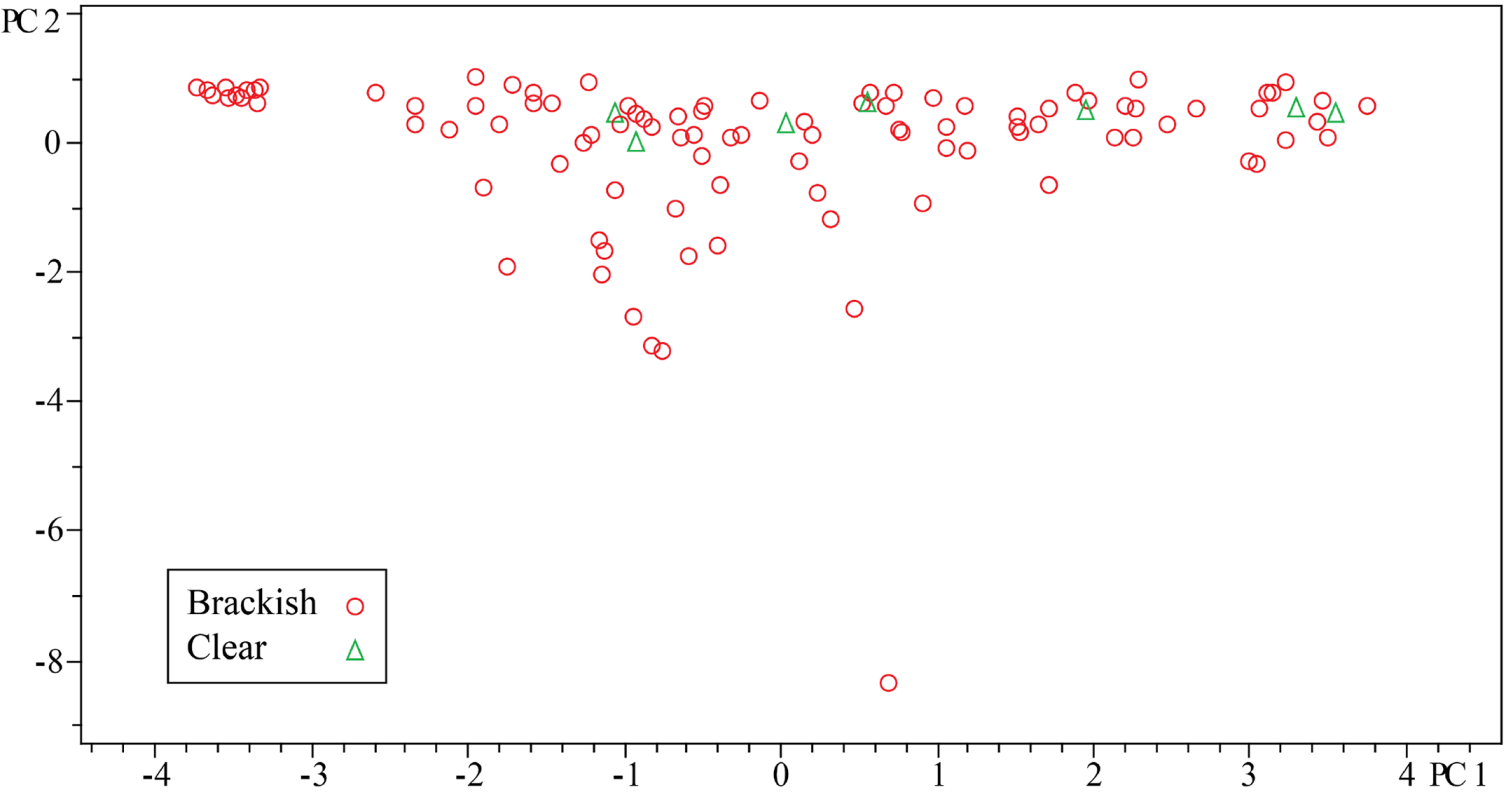
PCA scores plot for *Micromonospora* spp.

Since the PLS-DA indicated that much of the grouping could be defined by the genus and the genera appeared to be fairly specific, we next sought to analyze some of the other genera in a similar manner to our *Streptomyces* analysis. First, we wanted to understand if *Micromonospora* spp. from the clear habitat contained any unique chemistry as compared to the brackish habitat. For all Micromonosporaceae, strains were grown in the presence of iron and HP20 resin. The HP20 resin increases metabolite production, and the iron was added to reduce production of iron chelators related to desferrioxamine, which would skew the metabolomics evaluation. Initial analysis of all *Micromonospora* spp. by PCA indicated that seven strains isolated from the clear habitat did not add any unique chemistry to strains isolated from the brackish habitat. However, we noticed that when PCA was used to analyze a large number (105) of *Micromonospora* spp., the plot of PC1 versus PC2 showed one strain that was significantly different from the rest, but little information could be obtained regarding the remainder of strains. Mainly, the remaining strains appeared as a continuum across PC1 indicating that intergroup covariance was similar to intragroup covariance (Fig 8). Therefore, we sought a method that could help confirm or deny our conclusion based on PCA regarding *Micromonospora* spp. metabolic diversity between the two habitats.

**Fig 9:**
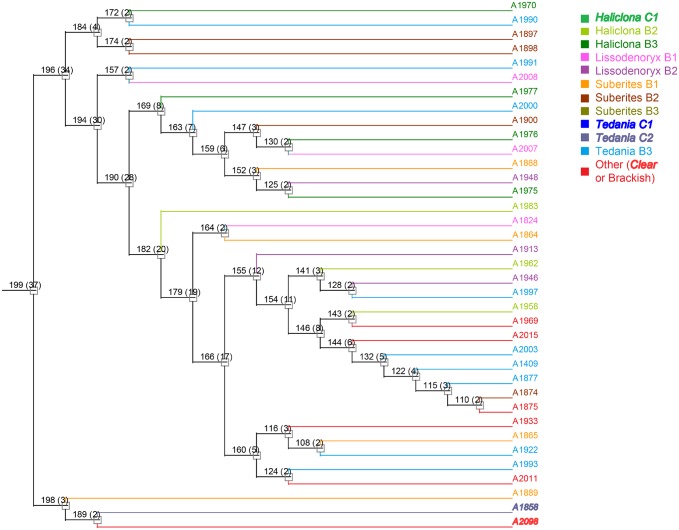
Representative sample of the hierarchal cluster of *Micromonospora* spp. Isolates collected from sponges of the same genera share similar colors, and clear tropical samples are represented in bold and italic formatting. Full tree is available in S2 Fig.

While DNA based phylogenetic methods for comparing bacteria are well known, comparing bacteria in a similar manner based on metabolites had not been done. While we and others have demonstrated that approaches such as PCA are well suited for identifying variance among metabolite profiles, PCA does not provide meaningful results when data sets become too complex, as seen above. Therefore, we evaluated methods for hierarchal cluster analysis (HCA) based on data from LCMS (Fig 9).

HCA was much more informative for observing relationships among a large group of strains. On the basis of PCA, *Micromonospora* spp. from clear water did not add any apparent chemical diversity. On the other hand, HCA clearly showed that some clear water *Micromonospora* spp. formed a distinct clade indicating unique RT-*m/z* features. For example Strains A1858 and A2098 were not identified as being distinct in PCA, but appeared unique by HCA. Further analysis and overlay of LCMS chromatograms showed that these two strains were nearly identical to each other, but had distinct RT-*m/z* features. Overall, HCA was better able to resolve differences. In part, the dataset was too varied for PCA to be an appropriate tool. For comparison of large numbers of diverse strains HCA would, in most cases, be a better method.

Using HCA, we also investigated whether relationships could be observed among isolates collected from sponges of the same genera. Colors were used in the HCA trees to highlight the genera of sponge each bacterial isolate was collected from. Similar color were used for sponges of the same genera. Clear tropical sponge isolates were also distinguished from brackish sponge isolates. For *Micromonospora* spp., no meaningful relationships were observed among isolates collected from sponges of the same genera.

**Fig 10:**
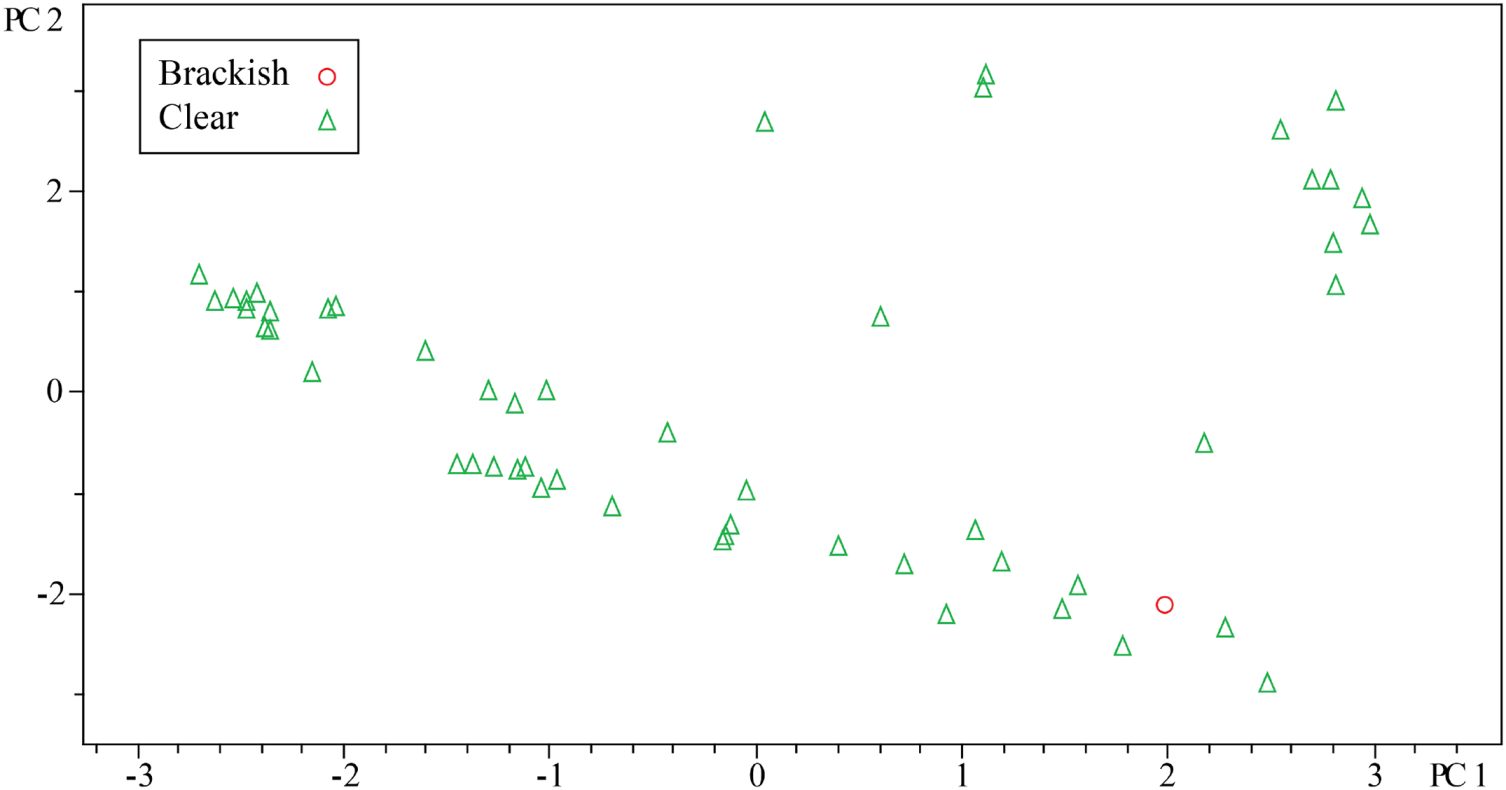
PCA scores plot for *Verrucosispora* spp.

**Fig 11:**
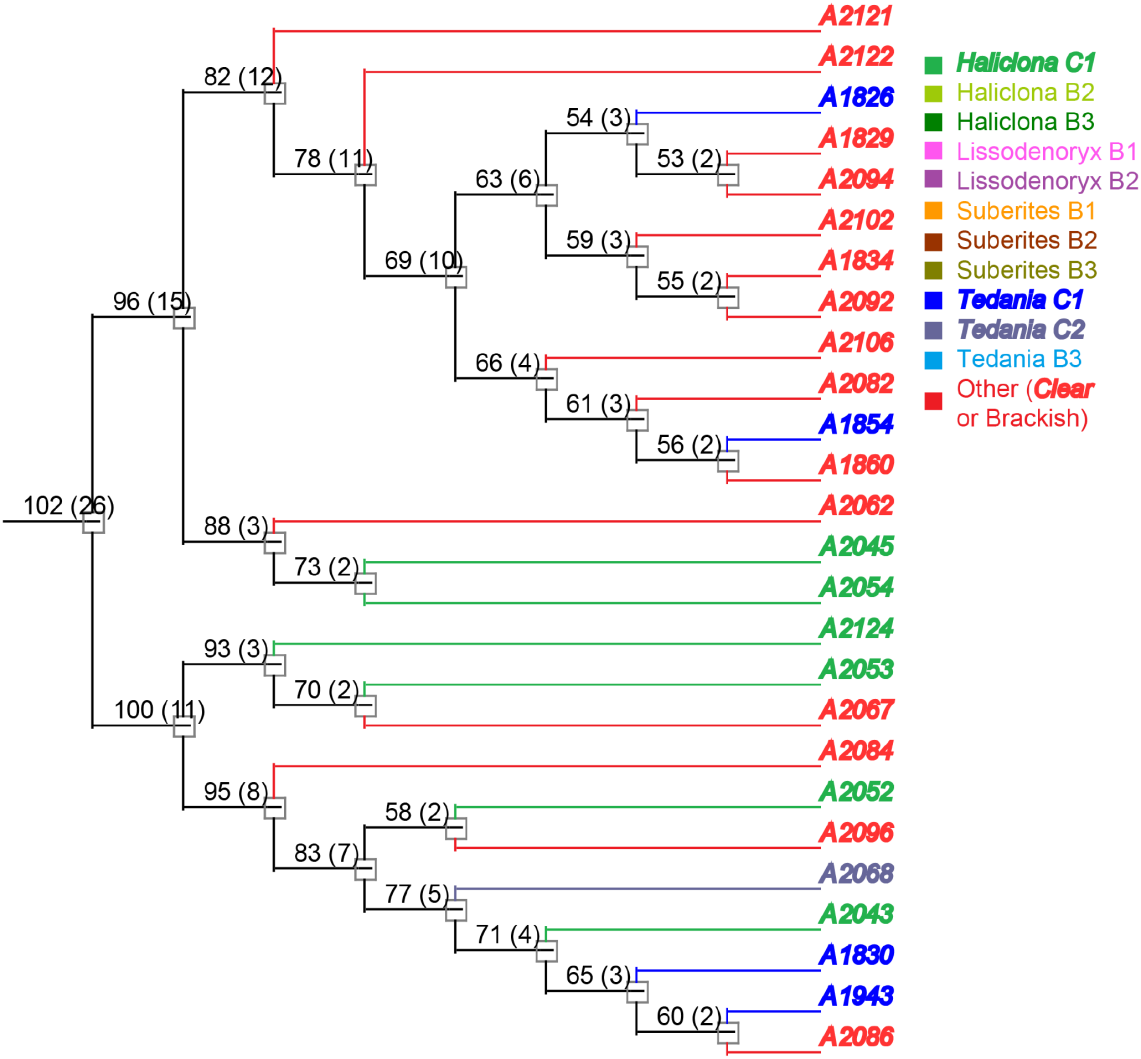
Representative sample of the hierarchal cluster of *Verrucosispora* spp. Isolates collected from sponges of the same genera share similar colors, and clear tropical samples are represented in bold and italic formatting. Full tree is available in S3 Fig.

Next, we investigated *Verrucosispora*, an actinomycete genus that appears to be fairly specific for the marine environment. *Verrucosispora* spp. have previously been cultivated from marine sponges in the China Seas.[37] Interestingly, only one *Verrucosispora* sp. was cultivated from the sponges from the brackish habitat. On the other hand, *Verrucosispora* spp. were easily cultivated from the sponges collected in the clear tropical habitat. Therefore, comparison between clear and brackish habitats is not as informative, but information could still be derived regarding the diversity within clear samples. In terms of LCMS analysis, the *Verrucosispora* spp. were chemically diverse indicating that additional sampling would be valuable for this genus. We did not observe relationships that correlated with the sponge genus in the HCA for the *Verrucosispora* spp. (Figs 10 and 11) Of course, this could indicate that more sampling was required or that *Verrucosispora* spp. were not specific, but casual associates.

### Bioassays

We also assessed all strains for biological activity using inhibition of *E*. *coli* and *B*. *subtilis*. In general we observed more activity among the strains isolated from the clear tropical habitat. Unfortunately, the numbers of strains isolated were also lower from that habitat; so, the data were not easily compared. Overall, however, we observed that 68% of the strains showed antimicrobial activity (Table 1).

**Table 1:** Biological activity data.

Genus	Clear Bioactive / Total	Brackish Bioactive / Total
*Streptomyces*	7 / 7	19 / 48
*Micromonospora*	5 / 7	68 / 98
*Verrucosispora*	45 / 53	0 / 1
*Solwaraspora*	14 / 18	0 / 0

## Conclusions

Overall, we found that sponges from the brackish environment yielded higher phylogenetic diversity of Actinobacteria over the tropical reef habitat. However, the brackish environment yielded fewer members of genera that have been classified as having specificity for the marine environment. For the purposes of broadly analyzing large numbers of strains by metabolomics, HCA provided better resolution and was much more informative. PCA works well for strain dereplication and selection among small groups of closely related strains, but as the variance increases, the ability to discriminate classes or groups declines. In terms of biological activity, little could be concluded. The use of PLS-DA assisted in understanding differences among bacteria from brackish versus the clear tropical habitat. Overall, the use of metabolomics helped define collection strategies for Actinomycetes from sponges and showed that the brackish environment was particularly rich in *Streptomyce*s spp. and *Micromonospora* spp. while the clear tropical habitat was particularly rich in *Verrucosispora* spp. We anticipate that the methods we have set forth could find broad use as a screen for improving collection strategies among a range of habitats and genera.

## Supporting information

S1 Fig Full hierarchal cluster of *Streptomyces* spp. Isolates collected from sponges of the same genera share similar colors, and the clear tropical samples are represented in bold and italic formatting.
(PDF)

S2 Fig Full hierarchal cluster of *Micromonospora* spp. Isolates collected from sponges of the same genera share similar colors, and the clear tropical samples are represented in bold and italic formatting.
(PDF)

S3 Fig Full hierarchal cluster of *Verrucosispora* spp. Isolates collected from sponges of the same genera share similar colors, and the clear tropical samples are represented in bold and italic formatting.
(PDF)

S4 Fig Phylogenetic tree of all bacterial isolates used in this analysis. (PDF)

S1 Table Taxonomy of sponge specimens. (XLSX)

S2 Table Accession numbers for bacterial isolates. (XLSX)
