# Supplementary figures and images for: Brackish habitat dictates cultivable Actinobacterial diversity from marine sponges
Source: PLoS One. 2017 Jul 10;12(7):e0176968. doi: 10.1371/journal.pone.0176968 (PMC5503172; doi:10.1371/journal.pone.0176968)

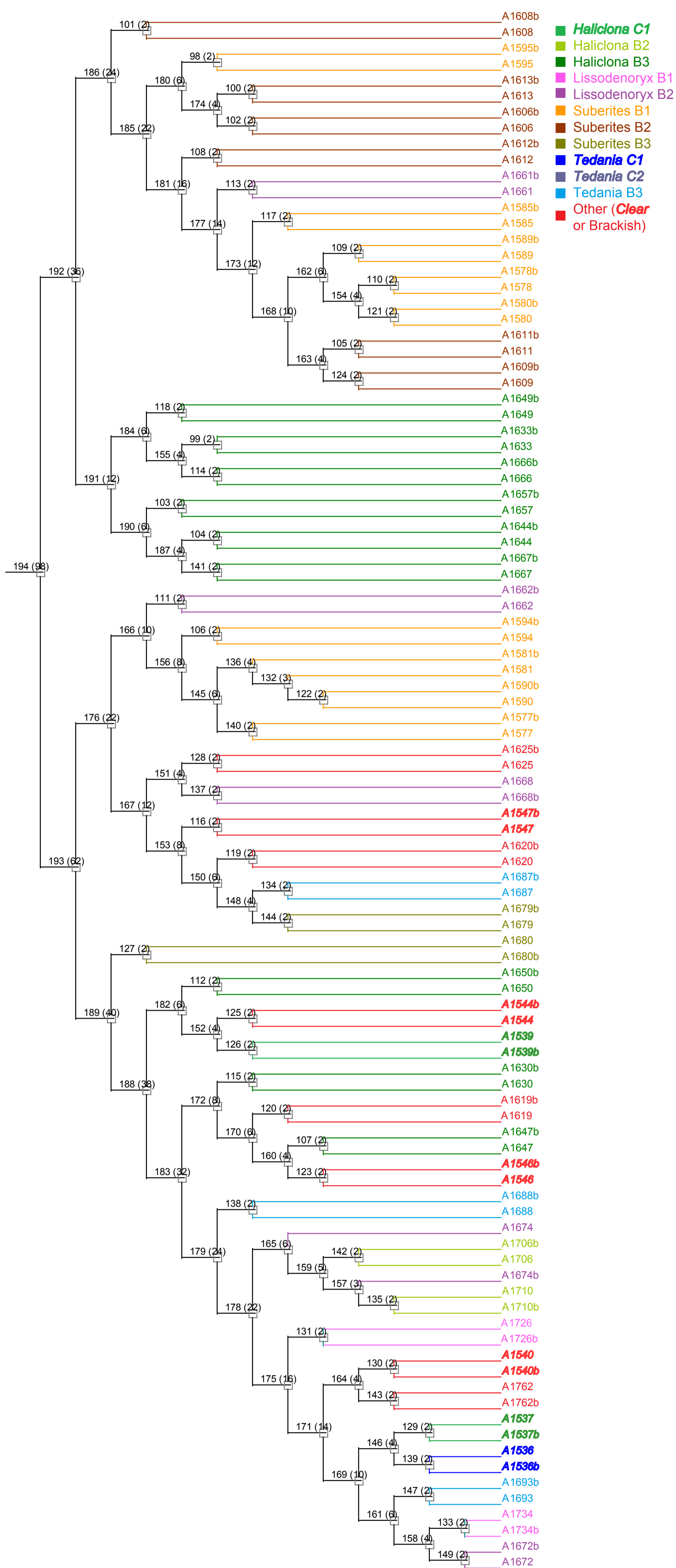

Supplement: S1 Fig — Isolates collected from sponges of the same genera share similar colors, and the clear tropical samples are represented in bold and italic formatting. (PDF) [file pone.0176968.s001.pdf]

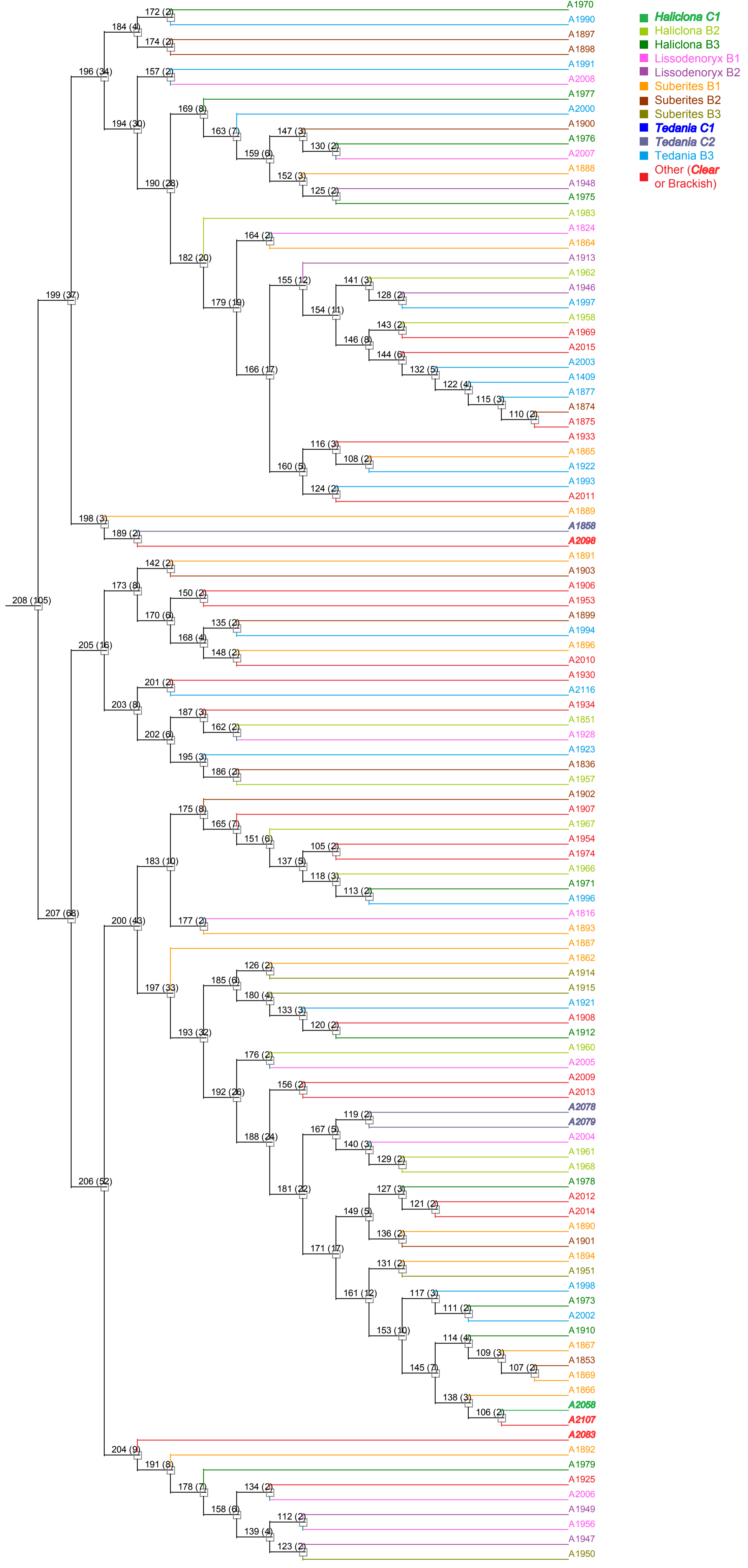

Supplement: S2 Fig — Isolates collected from sponges of the same genera share similar colors, and the clear tropical samples are represented in bold and italic formatting. (PDF) [file pone.0176968.s002.pdf]

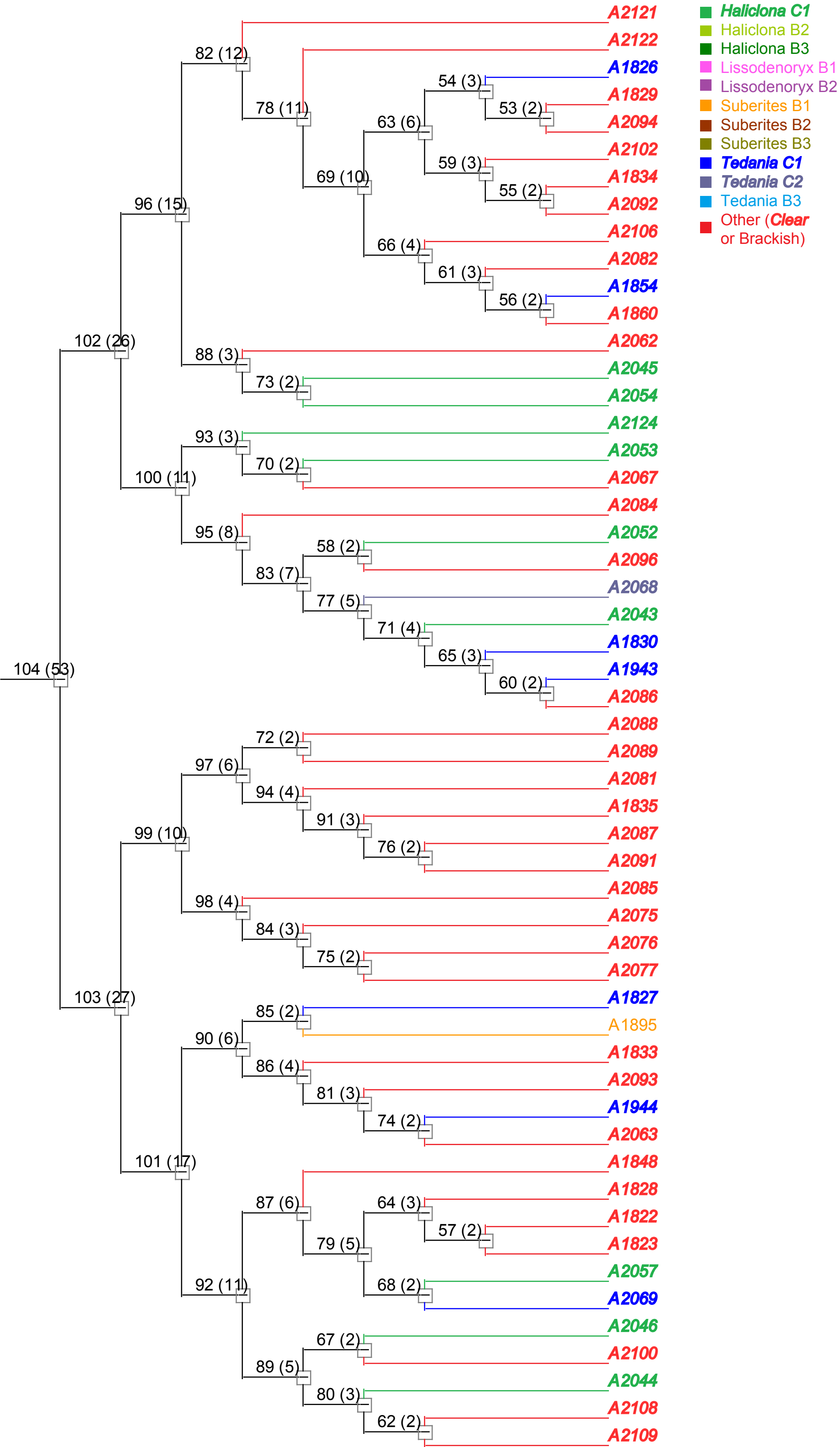

Supplement: S3 Fig — Isolates collected from sponges of the same genera share similar colors, and the clear tropical samples are represented in bold and italic formatting. (PDF) [file pone.0176968.s003.pdf]

Tree scale: 0.01

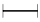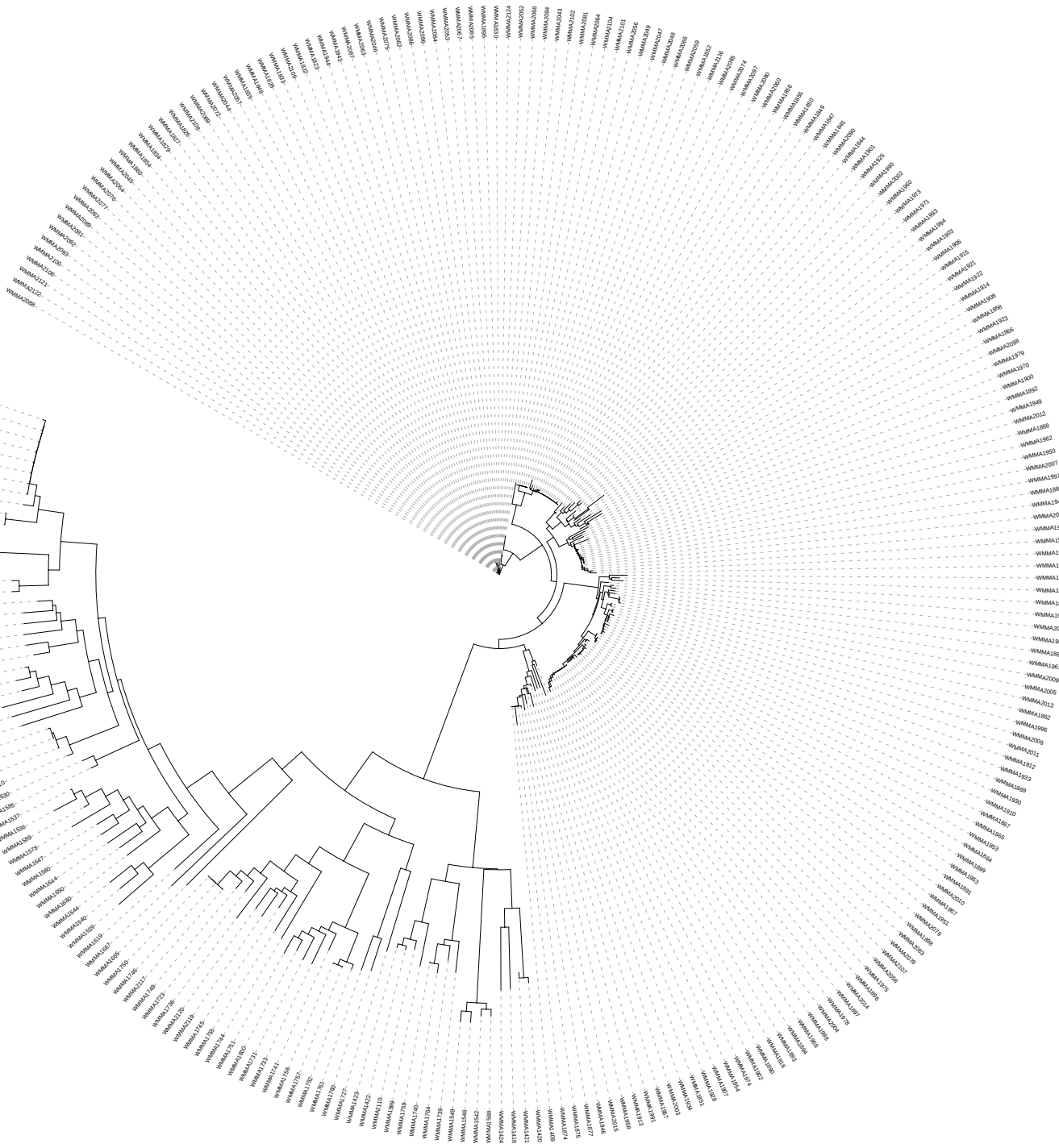

Supplement: S4 Fig — (PDF) [file pone.0176968.s004.pdf]
